# Supplementary material for: Is it time to change the approach of mental health stigma campaigns? An experimental investigation of the effect of campaign wording on stigma and help-seeking intentions
Source: PLoS One. 2022 Aug 18;17(8):e0273254. doi: 10.1371/journal.pone.0273254 (PMC9387789; doi:10.1371/journal.pone.0273254)
Supplement: Attachment — Submitted filename: Response to Reviewers1.docx [file pone.0273254.s002.docx]

Response to Reviewers

**Manuscript Title:** Is it time to change the approach of mental health stigma campaigns? An experimental investigation of the effect of campaign wording on stigma and help-seeking intentions.

We thank the reviewers for their thoughtful and important comments. We are pleased to have the opportunity to revise our paper in light of their feedback. We have made changes to our manuscript using tracked changes. Our response letter is formatted with the reviewers’ comments given in bold, our response in normal typeface, and where needed, extracts from the paper italicised.

# Editor Comments:

## 1. Please ensure that your manuscript meets PLOS ONE's style requirements, including those for file naming. The PLOS ONE style templates can be found at  <https://journals.plos.org/plosone/s/file?id=wjVg/PLOSOne_formatting_sample_main_body.pdf> and <https://journals.plos.org/plosone/s/file?id=ba62/PLOSOne_formatting_sample_title_authors_affiliations.pdf>

We have reviewed the guidance and have updated the manuscript to adhere to this guidance.

## 2. Please provide additional details regarding participant consent. In the ethics statement in the Methods and online submission information, please ensure that you have specified what type you obtained (for instance, written or verbal, and if verbal, how it was documented and witnessed). If your study included minors, state whether you obtained consent from parents or guardians. If the need for consent was waived by the ethics committee, please include this information.

We have included a statement to the ethics section of the method describing how consent was taken:

*Participants provided online informed consent by completing a tick box form.*

## 3. We note that you have indicated that data from this study are available upon request. PLOS only allows data to be available upon request if there are legal or ethical restrictions on sharing data publicly. For more information on unacceptable data access restrictions, please see <http://journals.plos.org/plosone/s/data-availability#loc-unacceptable-data-access-restrictions>. In your revised cover letter, please address the following prompts:

## a. If there are ethical or legal restrictions on sharing a de-identified data set, please explain them in detail (e.g., data contain potentially sensitive information, data are owned by a third-party organization, etc.) and who has imposed them (e.g., an ethics committee). Please also provide contact information for a data access committee, ethics committee, or other institutional body to which data requests may be sent.

## b. If there are no restrictions, please upload the minimal anonymized data set necessary to replicate your study findings as either Supporting Information files or to a stable, public repository and provide us with the relevant URLs, DOIs, or accession numbers. For a list of acceptable repositories, please see <http://journals.plos.org/plosone/s/data-availability#loc-recommended-repositories>.

## c. We will update your Data Availability statement on your behalf to reflect the information you provide.

We are happy to share the minimal anonymised data set from this study. Thank you for updating the data availability statement as required.

## 4. PLOS requires an ORCID iD for the corresponding author in Editorial Manager on papers submitted after December 6th, 2016. Please ensure that you have an ORCID iD and that it is validated in Editorial Manager. To do this, go to ‘Update my Information’ (in the upper left-hand corner of the main menu), and click on the Fetch/Validate link next to the ORCID field. This will take you to the ORCID site and allow you to create a new iD or authenticate a pre-existing iD in Editorial Manager. Please see the following video for instructions on linking an ORCID iD to your Editorial Manager account: <https://www.youtube.com/watch?v=_xcclfuvtxQ>

The corresponding author has an ORCID iD that can be attached to this submission: 0000-0001-58689902; however, I have been unable to do this when submitting and resubmitting the paper. I receive an error message each time I try to connect the ORCID iD. Any assistance you can provide in fixing this would be appreciated.

## 5. According to the journal's guideline, the authors need to conform to appropriate reporting guidelines (e.g. CONSORT for this study): <https://www.equator-network>.

This paper has been written in line with the CONSORT-SPI 2018 Extension guidelines. This has been included in the methods section.

# Reviewer 1 Comments:

## 6. Overall this is a well written paper on an important topic. The language is accessible and clear throughout. I’ve made a couple suggestions for how the authors may like to develop the paper, but these really are suggestions rather than requests. Thank you, it was a pleasure to read your paper. I recommend it’s publication.

We would like to thank the reviewer for their kind comments about our paper.

## 7. Abstract: The abstract is provides a clear description of the need for research, the methods used, the results found, and possible interpretations. At the moment the abstract is long and might benefit from being condensed to key points.

We have now revised the abstract to reduce its length. The abstract is now below 250 words.

## 8. Introduction: Excellent description of the need to challenge mental health-related stigma, current dominant strategies (e.g. TTC campaign), and the potential for advancing alternative approaches.

We would like to thank the reviewer for their kind comments about our paper.

## 9. Introduction: The paper, and subsequent research hypotheses, would benefit from engaging with theories of health communication to explain the theoretical model (e.g. Theory of planned behaviour; health belief model; social representations theory etc) by which visual communication is considered to influence individual attitudes.

We have included a sentence to the introduction to give a theoretical basis for the use of poster in anti-stigma campaigns:

*Anti-stigma campaigns have therefore tried to reduce public stigma towards people with mental health problems. These campaigns make use of visual representations to portray messages. Images are an effective way of communicating mental health-related information as they are thought to encourage elaborative thinking (Lazard et al., 2016).*

## 10. Methods: Clear description of research design, participants, and posters. Measures are well described and relevant. Clear account of analysis plan; relevant choice of MANCOVA for addressing increased Type I error.

We would like to thank the reviewer for their kind comments about our paper.

## 11. Results: Clear descriptive statistics of the sample characteristics and research data; and researchers effectively control for covariates.

We would like to thank the reviewer for their kind comments about our paper.

## 12. Discussion: Slight issues with wording on line 230: researchers states “The null findings may suggest the posters have equivalent effects, but are likely a result of the brevity of our intervention” as this statement was not directly evidenced, it might be more approach to say “may reflect the brevity” rather than “likely”.

We have amended the wording as follows:

*The null findings may suggest the posters have equivalent effects, but may also reflect the brevity of our intervention.*

## 13. Discussion: Interesting and balanced discussion of the ‘fear effect’. These is some research e.g. Thibodeau & Peterson (2018) that continuum-belief interventions to increase participants experiences of anxiety and threat. There is also evidence e.g. Foster, 2001; Walsh & Foster, 2020 that perceiving the Other as relevant to the Self is experienced as threatening for one’s social identity (and empowered position in the moral order).

We have amended this section of the discussion to include some of the literature described by the reviewer:

*For example, while believing people with mental health problems are dangerous and blameworthy are both stigmatising attitudes, their effects on help-seeking are distinct. The former of these attitudes (danger beliefs) is associated with increased help-seeking while the latter (personal responsibility beliefs) is associated with reduced help-seeking (Mojtabai, 2010). It is therefore possible that mental health stigma campaigns may produce effects on individual stigmatising attitudes. Assuming this is correct, the present non-categorical poster may have had a specific impact on fear-related beliefs by enhancing the perceived proximity of those with mental health problems. That is, people with mental health problems are not “other” but are instead part of their in-group. Being ‘close’ to someone with mental health problems may therefore specifically increase the perceived likelihood of threat resulting in increased fear (Thibodeau & Peterson, 2018). Our results here may reflect the adverse consequences of contact on specific mental health related attitudes.*

## 14. Implications: To support the statement on line 275 - 277: “On reflection, it is unlikely that such a simple intervention is likely to bring about significant and sustained changes in a construct as complex and multifaceted as stigma” It might be worth also highlighting that representations of mental health problems are historically tenacious (Jodelet, 1991) and very much embedded in institutions (e.g. the media) (Rose, 1998).

We have added a sentence to the discussion to reflect on the longstanding and pervasive nature of mental health stigma:

*On reflection, it is unlikely that such a simple intervention is likely to bring about significant and sustained changes in a construct as complex and multifaceted as stigma. Negative depictions of mental health have been prevalent for a long period of time (Schomerus & Angermeye, 2017) and are embedded both implicitly and explicitly in mainstream media (e.g. newspapers and magazines (Nawková et al., 2012) and television (Henderson, 2018)).*

# Reviewer 2 Comments:

## 15. Introduction: The summary of the impact of Time to Change is based on results published part way into the campaign, which finished in 2021. For the results published towards the end ie after more changes had accrued, see Henderson C, Potts L, Robinson EJ. Mental illness stigma after a decade of Time to Change England: inequalities as targets for further improvement. European Journal of Public Health, 30 (3): 526–532, <https://doi.org/10.1093/eurpub/ckaa013>, 2020.

Thank you to the reviewer for highlighting this reference. We have added it to our introduction:

*TTC was launched in 2009 and evaluations have generally shown incremental improvements in reducing public stigma around mental health with each year of its existence (Evans-Lacko et al., 2014; Henderson et al., 2020; Sampogna et al., 2017).*

## 16. Introduction: The discussion about a categorical vs noncategorial approach needs more work. First, emphasising the prevalence of mental health problems is a way to normalise them and raise people’s awareness of common mental disorder, the symptoms of which are at the severe end of experiences that everyone has at some point, therefore the use of a prevalence message is not purely in opposition to a noncategorical one.

We appreciate that prevalence statistics and the categorical approach to mental health are not necessarily interchangeable. We have included further information about how we do believe in this instance the “1 in 4” message does communicate a categorical understanding of mental health problems:

*TTC mirrored this approach in their frequent mention of clinical diagnoses and the delineation of those with and without mental health problems in their primary tagline of “1 in 4 people will experience a mental health problem in their lifetime”. Such prevalence statistics describe the number of people with versus without a particular symptom or characteristic (National Institute of Mental Health (NIMH), 2022) – therein implying a categorical perspective of mental health with one person having a mental illness, while the other three do not.*

## 17. Second, the body of work by Georg Schomerus and others on the continuum model is entirely neglected; instead the hypotheses are presented as though no one had tested them before.

We have added references to the work of Georg Schomerus and colleagues within the introduction and used the latest work from this group to help put our study into context:

*In a recent review, the opposing perspective of a non-categorical approach (also referred to as the continuum approach) was shown to generally be associated with a reduction in mental health stigma (Peter et al., 2021). But while this approach may be superior in reducing stigma, it may have some unintended negative consequences. There is some limited literature suggesting that over-normalising mental health problems (i.e. removing any notion of “otherness”) can adversely impact help-seeking (Biddle et al., 2007; Fernandez et al., 2022).*

We also referred to this work in order to establish the rationale for our project:

*The review by Peter et al. (2021) brought together the findings of 8 intervention studies, three of which were with members of the public. However, Peter et al. (2021) highlights these studies are limited in that they did not manipulate the intervention message and therefore cannot provide any causal evidence of their impact of non-categorical beliefs on stigma. To our knowledge, there is currently no experimental test of the impact of a categorical versus non-categorical anti-stigma campaign that assesses its impact on both stigma and help-seeking outcomes.*

## 18. Methods: As this is an online RCT I recommend the use of the online RCT extension of the Consort reporting guidance, including a consort flow diagram.

Based on a search of the EQUATOR network guidelines, we have decided to use the CONSORT-SPI 2018 Extension guidelines as this is the best fit for experimental studies in the area of psychology.

## 19. Method: The categorical poster description of diagnosis/problem appears to assume that these are synonymous in people’s minds. This is not the case- many people think of nondiagnostic issues as mental health problems. This is something that increased over Time to Change, the evaluation of which included assessment of whether people think of grief and stress as mental health problems.

We have made some changes to the wording in how we describe the posters to make it clear that the categorical approach refers to there being a boundary delineating good and poor mental health, and that this may, but not necessarily, reflect the presence and absence of a psychiatric diagnosis:

*The categorical poster aligns with the medical model of mental health whereby good and poor mental health can be clearly delineated with cut-offs that describe the person as either having or not having a diagnosable mental health problem (American Psychiatric Association, 2013; World Health Organisation (WHO), 1992)… The non-categorical poster moves away from cut-offs and/or diagnostic labels and instead emphasises that we all have mental health and that this is fluctuating and changeable.*

## 20. Method: The AQ ask about people with mental health problems, implying a categorical attitude. Is this a problem for measurement of the impact of the noncategorical poster?

Thank you for raising this issue. We have acknowledged this within our limitations:

*Further issues related to our method of measurement is that the wording of the AQ may have tainted our experimental manipulation – especially for the non-categorical poster arm. The language used in the AQ may have subtly communicated an alignment with the categorical approach i.e. referring to people with mental health problems, suggesting an “otherness”. Participants completing this questionnaire in the non-categorical arm will have viewed potentially contradictory ideas and the impact of this on their responses cannot be fully determined. Addressing this in future research studies will be a challenge for researchers who must consider ways of measuring endorsement of competing ideologies using neutral language.*

## 21. Discussion: Given the evidence cited for the lack of impact of short-term campaigns on the outcomes of interest it is not clear what the rationale for the current study was.

We included additional text in the introduction that sets out the rationale for this project.

*The review by Peter et al. (2021) brought together the findings of eight intervention studies, three of which were with members of the public. However, Peter et al. (2021) highlights these studies are limited in that they did not manipulate the intervention message and therefore cannot provide any causal evidence of their impact of non-categorical beliefs on stigma. To our knowledge, there is currently no experimental test of the impact of a categorical versus non-categorical anti-stigma campaign that assesses its impact on both stigma and help-seeking outcomes.*

We acknowledge that short term anti-stigma campaigns have limited effectiveness but as this type of study had not been done before (i.e. an experimental test of categorical versus non-categorical anti-stigma campaigns that assess the impact on both stigma and help-seeking outcomes) we felt that using brief campaigns was most appropriate for this initial test of our hypotheses. We acknowledge the issues with brief anti-stigma campaigns in our discussion.

## 22. The implications section again ignores the existing body of work on the continuum model. How does the current study build on the work of Schomerus et al? Without such contextualisation and given the likely effect of a high prevalence message on reducing othering it is hard to see what this study adds.

We have amended the implications section of the discussion to make mention of the work of Schomerus and colleagues.

*Our null findings mean we cannot offer any suggestion as to which approach, categorical or non-categorical, is most effective at reducing mental health stigma and encouraging help-seeking when needed. Previous literature suggests a superiority of the non-categorical (continuum) approach, but these intervention studies are limited by their use of cross-sectional rather than experimental designs (Peter et al., 2021). We therefore do not yet know if there is an approach to anti-stigma campaigns that can surpass the gains achieved by the Time to Change approach.*

We thank the reviewers for taking the time to review our paper and for offering constructive comments to strengthen it. Please do not hesitate to contact a member of our team if any of the points require further clarification.

Best wishes,

Dr Cassie M Hazell (on behalf of the wider research team).
