# Supplementary material for: Is it time to change the approach of mental health stigma campaigns? An experimental investigation of the effect of campaign wording on stigma and help-seeking intentions
Source: PLoS One. 2022 Aug 18;17(8):e0273254. doi: 10.1371/journal.pone.0273254 (PMC9387789; doi:10.1371/journal.pone.0273254)
Supplement: Attachment — Submitted filename: Response to Reviewers 19.07.2022.docx [file pone.0273254.s003.docx]

Response to Reviewers

**Manuscript Title:** Is it time to change the approach of mental health stigma campaigns? An experimental investigation of the effect of campaign wording on stigma and help-seeking intentions.

We thank the reviewers for their thoughtful and important comments. We are pleased to have the opportunity to revise our paper in light of their feedback. We have made changes to our manuscript using tracked changes. Our response letter is formatted with the reviewers’ comments given in bold, our response in normal typeface, and where needed, extracts from the paper italicised.

# Reviewer 1 Comments:

## 1. I recommend it's publication. It is a well written article on an important topic. It was already of a high quality. The authors responded diligently to both my and the other reviewer’s comments. It was a pleasure to review.

We would like to thank the reviewer for their kind comments about our paper.

# Reviewer 2 Comments:

## 2. Regarding the authors' response: “To our knowledge, there is currently no experimental test of the impact of a categorical versus non-categorical anti-stigma campaign that assesses its impact on both stigma and help-seeking outcomes.” The authors did not assess help seeking outcomes. The abstract begins accurately by using the term help seeking intentions, and then changes to help seeking outcomes. Please correct this. Once this is accurate, the rather minimal difference in outcomes used between this study and doi: 10.1016/j.eurpsy.2015.11.006 becomes apparent; the overemphasis of the novelty of the study then still needs to be addressed.

We respect the feedback from the reviewer and appreciate the opportunity to improve our paper. The use of ‘help-seeking outcomes’ to describe our dependent variable is inappropriate and we have revised all instance of this to ‘help-seeking intentions’.

With regard to the novelty of our paper, we do agree that there are similarities with the paper by Schomerus et al. (2016). Both papers are comparing continuum versus dichotomous approaches to mental health on mental health on mental health stigma. We do however posit that our paper is distinct from the work by Schomerus et al. (2016) in that we also assessed help-seeking intentions. The paper by Schomerus et al. (2016) does not assess this construct (the measures included are the Social Distance Scale, continuum and difference beliefs, and stereotype endorsement). We would therefore like to request that the current rationale remains as presented in the revised version of the manuscript.

We thank the reviewers for taking the time to review our paper and for offering constructive comments to strengthen it. Please do not hesitate to contact a member of our team if any of the points require further clarification.

Best wishes,

Dr Cassie M Hazell (on behalf of the wider research team).
